# Photon-Counting Detector CT Allows Abdominal Virtual Monoenergetic Imaging at Lower Kiloelectron Volt Level with Lower Noise Using Lower Radiation Dose: A Prospective Matched Study Compared to Energy-Integrating Detector CT

**DOI:** 10.1007/s10278-025-01593-5

**Published:** 2025-07-02

**Authors:** Huan Zhang, Yangfan Hu, Lingyun Wang, Yue Xing, Zhihan Xu, Junjie Lu, Jiarui Yang, Bei Ding, Fei Yuan, Jingyu Zhong

**Affiliations:** 1 Department of Radiology, Ruijin Hospital, Shanghai Jiao Tong University School of Medicine, Shanghai, 200025 China; 2 Department of Imaging, Tongren Hospital, Shanghai Jiao Tong University School of Medicine, Shanghai, 200336 China; 3 Shanghai Key Laboratory of Flexible Medical Robotics, Institute of Medical Robotics, Tongren Hospital, Shanghai Jiao Tong University, Shanghai, 200336 China; 4 Siemens Healthineers, Shanghai, 201318 China; 5 Department of Epidemiology and Population Health, Stanford University School of Medicine, Stanford, CA 94305 USA; 6 Department of Biomedical Engineering, Boston University, Boston, MA 02215 USA; 7 Department of Pathology, Ruijin Hospital, Shanghai Jiao Tong University School of Medicine, Shanghai, 200025 China

**Keywords:** Multidetector computed tomography, Image reconstruction, Image enhancement, Radiation dosage

## Abstract

Our study aimed to assess the image quality of lower kiloelectron volt (keV) level abdominal virtual monoenergetic imaging (VMI) with lower radiation dose on photon-counting detector computed tomography (PCD-CT), in comparison to energy-integrating detector computed tomography (EID-CT). We prospectively included three matched groups, each with 59 participants, to undergo contrast-enhanced abdominal CT scans using EID-CT with full-dose (EID_FD), PCD-CT with full-dose (PCD_FD), and PCD-CT with low-dose (PCD_LD) protocols, respectively. The data of portal-venous phase were reconstructed into VMI at 40, 50, 60, and 70 keV, respectively. The standard deviation of CT values in liver parenchyma was measured as image noise. The signal-to-noise ratio (SNR) of liver parenchyma and contrast-to-noise ratio (CNR) of liver-portal vein were calculated. Three radiologists assessed the image noise, vessel sharpness, and overall quality, and rated the hepatic lesion conspicuity if possible. Our study found that the PCD_LD significantly reduced the radiation dose than EID_FD or PCD_FD (*p* < 0.001). The noise was significantly decreased by PCD_FD and PCD_LD compared to EID_FD, but SNR values were significantly increased (*p* ≤ 0.006). The CNR values were significantly increased by PCD_FD and PCD_LD compared to EID_FD in VMI at 40 keV and 50 keV (*p* ≤ 0.010). The ratings of image noise, vessel sharpness, overall quality, and lesion conspicuity were significantly greater in PCD_FD and PCD_LD compared to EID_FD (*p* ≤ 0.001). There was no significant difference detected in rating of lesion conspicuity between PCD_FD and PCD_LD (*p* ≥ 0.259). In conclusion, PCD-CT allows abdominal VMI with lower keV and lower noise using lower radiation dose, to provide better visualization of the hepatic lesions.

## Introduction

Both dual-energy energy-integrating detector computed tomography (EID-CT) and photon-counting detector computed tomography (PCD-CT) allow virtual monoenergetic images (VMI) to simulate the appearance of a CT image at a specific monochromatic X-ray beam energy [1–3]. The VMI at low kiloelectron volt (keV) is useful in abdominal imaging, as it can improve iodine attenuation. Indeed, approaching the k-edge of iodine (33 keV) increases the attenuation of enhancing lesions and can increase visibility and contrast, but is partly mitigated by noise. The lesion conspicuity may vary based on the scanner platform and noise mitigation strategies in that particular system. For dual-energy EID-CT, the standard keV level for VMI varies based on the specific scanner platform and institutional preferences. It can be 50 to 60 keV in abdomen portal-venous phase scans using iterative reconstruction algorithms in some cases [4–7], while reaching a lower keV level of 40 keV with an acceptable image quality needs the help of deep-learning based reconstruction algorithms [8–10]. To reduce image noise and increase image quality, the advanced PCD-CT scanners yield direct conversion of incoming photons into electronic signals and count each individual X-ray photon in the scan [11–13]. As the PCD-CT no longer suffers from the electronic noise, the PCD-CT system has lower image noise and allows VMI at 40 to 50 keV to have acceptable image quality [14–16].

Further, as PCD-CT can guarantee image quality and acceptable image noise with appropriate reconstruction settings even with lower radiation dose, an extra radiation dose reduction may be possible. The PCT-CT can generate VMI at 40 to 50 keV to have lower image noise and better image quality in comparison to dual-energy EID-CT with identical radiation dose in abdominal scans [14–16], indicating its potential in radiation dose reduction. In addition to the advantages in parenchymatous organs, PCD-CT has been demonstrated to enable lower keV VMI for better visibility of abdominal arteries with lower radiation dose and lower contrast dosage, and has been considered the optimal imaging method in comparison to dual-energy EID-CT [17–20]. The decreased image noise and better image quality have been translated into higher lesion conspicuity with lower radiation dose in comparison to dual-energy EID-CT [12, 13, 21, 22]. Therefore, we assumed that the PCD-CT may allow abdomen portal-venous phase scans to have acceptable image quality in lower keV VMI with optimal reconstruction settings even using a lower dose protocol. Considering the potential of PCD-CT, we decided to assess the clinical acceptance of VMI on PCD-CT at lower kiloelectron volt levels with lower noise using lower radiation dose.

In our study, we aim to assess the image quality of lower keV level abdominal VMI with lower radiation dose on PCD-CT, in comparison to EID-CT.

## Methods

### Study Design and Participants

**Fig. 1:**
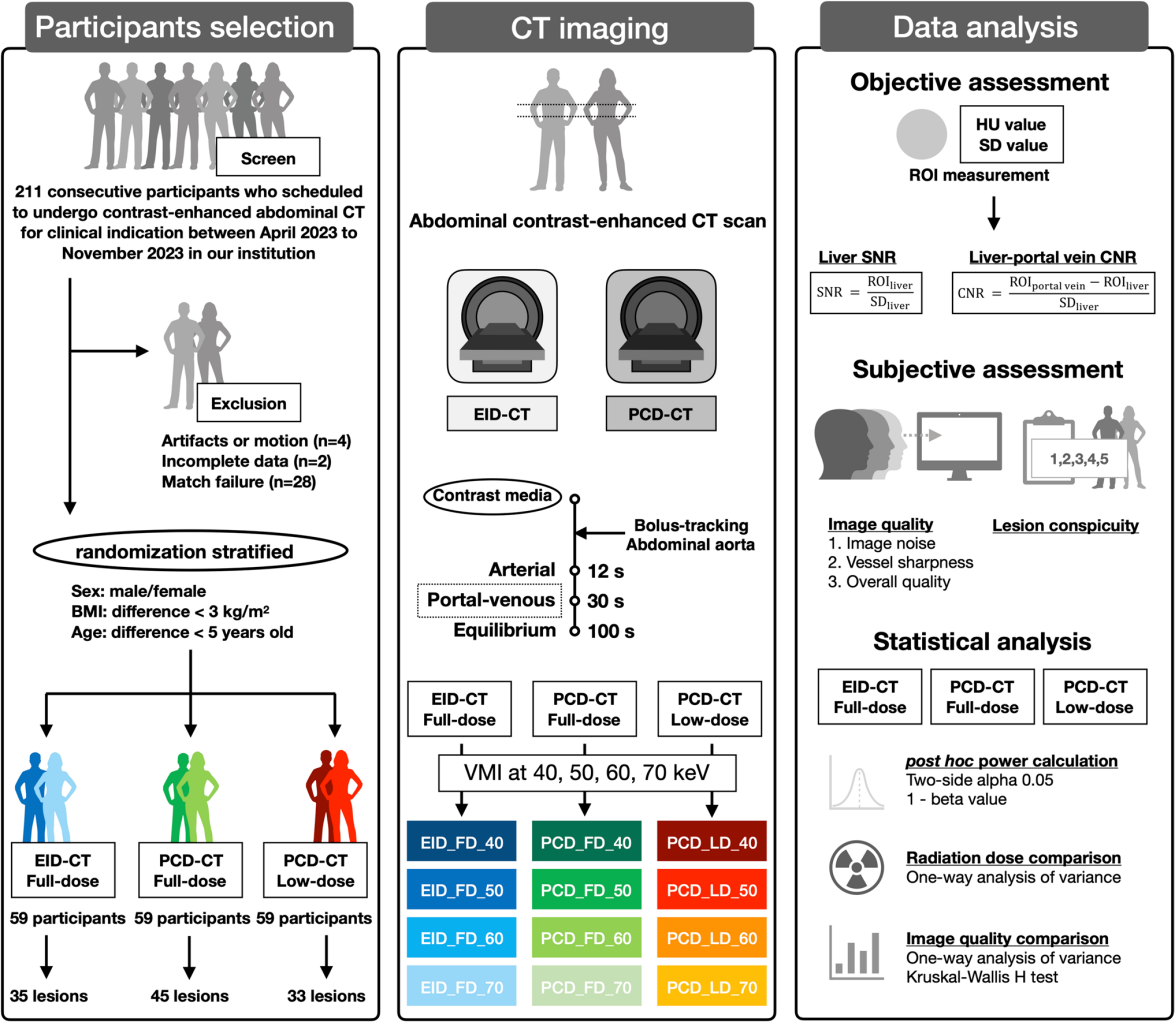
Study workflow. Our study included three steps: participants selection, CT imaging, and data analysis. EID_FD, scan on energy-integrating detector computed tomography using full-dose protocol; PCD-CT, scan on photon-counting detector computed tomography using full-dose protocol; PCD_LD, scan on photon-counting detector computed tomography using low-dose protocol; VMI, virtual monoenergetic imaging; keV, kiloelectron volt; HU, Hounsfield unit; SD, standard deviation; SNR, signal-to-noise ratio; CNR, contrast-to-noise ratio

This prospective study has been approved by the institutional ethical committee, and the written informed consents have been received from all the participants (Fig. 1). The participants who scheduled to undergo abdominal contrast-enhanced CT scans for clinical indication were scanned by a convenience sample between April 2023 and November 2023. The inclusion criteria were as follows: (a) age ≥ 18 years old; (b) without surgery history; and (c) agree to participate and give the written informed consent. The exclusion criteria were as follows: (a) severe artifacts or motion; (b) incomplete data; and (c) match failure. The participants were selected and divided into three groups that matched by sex, body mass index (less than 3 kg/m^2^), and age (less than 5 years) [23, 24]. Once there were a group of three patients meeting the criteria, they would be stratified into three different groups.

### Image Acquisition and Reconstruction

The participants were scanned on an EID-CT (SOMATOM Force, Syngo.CT version VB20_A, Siemens Healthineers) with a full-dose protocol (EID_FD), or a PCD-CT (NAEOTOM Alpha, Syngo.CT version VA50-SP1, Siemens Healthineers) with a full-dose protocol (PCD_FD) or a low-dose protocol (PCD_LD) (Table 1). The EID_FD scans were performed on the EID-CT scanner according to the optimized daily routine full-dose protocol with a fixed tube voltage of Sn150/90 kVp with automatic tube current modulation (CARE Dose 4D, Siemens Healthineers) using 125/200 mA as reference tube currents [25–27]. The PCD_FD and PCD_LD scans were performed using a fixed tube voltage of 140 kVp within automatic tube current modulation (CARE Dose 4D, Siemens Healthineers) at an image quality level of 145 and 90 for full-dose and low-dose scans on the PCD-CT scanner, respectively. All the patients received the same contrast administration protocol. The nonionic contrast material (1.5 mL/kg body weight, Ultravist 370, Schering) were administrated at a rate of 3.0 mL/s using a pump injector. The portal-venous phase scan was initiated with a 30-s delay when the abdominal aorta enhancement appeared.

**Table 1:** Acquisition and reconstruction parameters

	EID_FD	PCD_FD	PCD_LD
Scanner	SOMATOM Force	NAEOTOM Alpha	NAEOTOM Alpha
Tube voltage, kVp	Sn150/90	140	140
Tube current modulation method	CARE Dose 4D with reference tube current of 125/200 mA	CARE Dose 4D with image quality level of 145	CARE Dose 4D with image quality level of 90
Collimation, mm	128 × 0.6	144 × 0.4	144 × 0.4
Rotation time, s	0.5	0.5	0.5
Pitch	0.6	0.8	0.8
FOV, mm	374 × 374	automatically adjusted	automatically adjusted
Matrix	512 × 512	automatically adjusted	automatically adjusted
Thickness, mm	1.0	1.0	1.0
Increment, mm	1.0	0.7	0.7
Reconstruction algorithm	ADMIRE 3/5	QIR 4/4	QIR 4/4
Reconstruction kernel	Qr40	Br40	Br40

*EID_FD* scan on energy-integrating detector computed tomography using full-dose protocol, *PCD-CT* scan on photon-counting detector computed tomography using full-dose protocol, *PCD_LD* scan on photon-counting detector computed tomography using low-dose protocol, *ADMIRE* advanced modeled iterative reconstruction, *QIR* quantum iterative reconstruction, *FOV* field of view

All the data was anonymously retrieved and reconstructed using a manufacturer-specific spectral workstation (Syngo.Via version VB60, Siemens Healthineers). The VMI at 40, 50, 60, and 70 keV were reconstructed with a slice thickness of 1 mm for three groups with optimized parameters for abdominal scans. The EID_FD scans were reconstructed using a fixed matrix and slice thickness/increment, with an iterative reconstruction algorithm within a strength level of 3 out of 5 using a Qr40 kernel [25–27]. The PCD_FD and PCD_LD scans were reconstructed using an automatically adjusted matrix but fixed slice thickness/increment, with a quantum iterative reconstruction (QIR) algorithm that was specifically designed for PCD-CT within a strength level of 4 out of 4 using Br40, which was recommended for abdomen reconstruction by the vendor and a previous study [28].

### Radiation Dose

The radiation dose in our institution for abdominal CT scans on EID-CT scanner was lower than the 25th percentile volume CT dose index (CTDIvol) reported by the American College of Radiology Dose Index Registry (10 mGy) [29] and the local standard (10 mGy) [30]. We set the parameters to approximate 66% radiation dose from the standard PCD-CT dose and approximately 75% radiation dose from the routine EID-CT dose in our institution. The volume dose index and dose-length product were directly extracted from dose reports that were automatically generated by the scanners. The effective radiation dose was calculated using dose-length product multiplied by a conversion factor for the abdomen (*k* = 0.015) [31].

### Objective Image Evaluation

The objective image evaluation was conducted by a radiologist with 6 years of experience (JYZ) using the manufacturer-specific spectral workstation (Syngo.Via version VB60, Siemens Healthineers) (Supplementary Note S1). Circular regions of interest (ROIs) were placed to measure the Hounsfield unit (HU) values and standard deviation (SD) values. The ROIs with sizes of approximately 1 to 5 cm^2^ were placed on the right anterior, right posterior, left inner, and left outer parts of liver parenchyma, avoiding confounding structures such as vessels or lesions, as well as on the portal vein. The average SD values of liver were considered background noise. The signal-to-noise (SNR) values were calculated as the SNR _liver_ = average HU _liver_/average SD _liver_. The contrast-to-noise (CNR) values were calculated as CNR _liver_ = (HU _portal vein_ – HU _liver_)/SD _liver_. The measurements were considered with excellent inter-rater and intra-rater agreement [9, 10, 32, 33].

### Subjective Image Evaluation

The subjective image evaluation was performed by three radiologists with 6, 6, and 7 years of experience (YFH, LYW, and YX), respectively, using monitors (MDCC-4430, BRACO Co. Ltd.) with the same settings employed for daily image interpretation at the reading room (Supplementary Note S2). The images were presented to the readers in a random fashion blinded to the reconstruction parameters, with a default window width of 800 HU and window level of 200 HU. The readers were allowed to adjust the window width and level and viewing distance as they preferred and had no time limits to complete the image review. The image noise, vessel sharpness, and overall quality were rated using a five-point Likert scale. The rating of less than 3 was deemed unsatisfactory for clinical diagnostic purposes. For hepatic lesion conspicuity, the readers were told which specific lesion to rate in each participant but had to detect them by themselves. The ratings were considered with moderate to excellent inter-rater and intra-rater agreement [9, 10, 32, 33].

### Statistical Analysis

The statistical analysis was carried out by an author with 6 years of experience in radiology research (JYZ) using R language version 4.1.3 (https://www.r-project.org/) within related packages within RStudio version 1.4.1106 (https://www.rstudio.com/). The continuous variables are presented as mean ± standard deviation or median (the first and the third quartile), while the categorical variables are presented as frequency (percentage), respectively. One-way analysis of variance and Kruskal–Wallis H test were used to analyze differences in objective and subjective assessments among three groups within the same keV level, respectively. If a significant difference was found, the post hoc pairwise comparisons using Bonferroni method were applied. The two-sided alpha level was set at 0.05. The post hoc power calculation was performed using sample size and obtained SNR and CNR values and overall quality ratings with a two-sided alpha level of 0.05 [34].

## Results

### Participant Characteristics and Radiation Dose

We included 59 participants for each group, in which 35, 45, and 33 hepatic lesions were detected for evaluation for EID_FD, PCD_FD, and PCD_LD groups, respectively (Table 2). The sample size of our study obtained 1-beta value > 0.999, when alpha level was 0.05, indicating the high statistical efficiency (Supplementary Note S3). There was no significant difference between three matched groups in sex, age, height, weight, and body mass index (all *p* > 0.999), but the PCD_LD showed significant radiation dose reduction compared to PCD_FD and EID_FD (all adjusted *p* < 0.001). There was no significant difference in the lesion size (*p* = 0.539) and attenuation of lesions to liver (*p* = 0.937). According to volume CT dose index, dose-length product, and effective dose, the PCD_LD realized 34.6 ± 9.9%, 33.8 ± 17.8%, and 33.8 ± 17.8% reduction compared to the PCD_FD, and 27.3 ± 13.4%, 27.8 ± 20.4%, and 27.8 ± 20.4% compared to the EID_FD, respectively.

**Table 2:** Characteristics of participants

	EID_FD	PCD_FD	PCD_LD	*p*	*p*1	*p*2	*p*3
Sex				> 0.999	N. a	N. a	N. a
Male	41 (69)	41 (69)	41 (69)				
Female	18 (31)	18 (31)	18 (31)				
Age, year	64.5 ± 9.3	65.1 ± 9.1	64.3 ± 10.2	> 0.999	N. a	N. a	N. a
Hight, m	1.65 ± 0.07	1.65 ± 0.08	1.66 ± 0.07	> 0.999	N. a	N. a	N. a
Weight, kg	62.1 ± 8.4	63.0 ± 9.9	63.0 ± 10.0	> 0.999	N. a	N. a	N. a
Body mass index, kg/m^2^	22.9 ± 2.6	23.0 ± 2.5	22.8 ± 2.7	> 0.999	N. a	N. a	N. a
Volume CT dose index, mGy	5.47 ± 1.26	5.99 ± 0.97	3.87 ± 0.62	< 0.001	0.040	< 0.001	< 0.001
Dose-length product, mGy·cm	338.6 ± 92.1	365.3 ± 83.3	234.4 ± 56.6	< 0.001	0.305	< 0.001	< 0.001
Effective dose, mSv	5.08 ± 1.38	5.52 ± 1.30	3.52 ± 0.85	< 0.001	0.229	< 0.001	< 0.001
Number of lesions	35	45	33	N. a	N. a	N. a	N. a
Largest diameter of lesions, mm	15 (12 to 48)	8 (6 to 53)	10 (5 to 47)	0.539	N. a	N. a	N. a
Attenuation of lesions to liver				0.937	N. a	N. a	N. a
Lower	31 (89)	37 (82)	29 (88)				
Higher	3 (9)	6 (13)	3 (9)				
Mixed	1 (3)	2 (4)	1 (3)				

Data were presented as frequency (percentage) or mean ± standard deviation. *p p* value for chi-square test or one-way analysis of variance among the EID_FD, PCD_FD, and PCD_LD groups. *p*1 to *p*3 were *p* values for post hoc pairwise comparisons using Bonferroni correction; the *p* values presented were presented as adjusted *p* values. *p1 p* value between EID_FD and PCD_FD groups, *p2 p* value between EID_FD and PCD_LD groups, *p3 p* value between PCD_FD, PCD_LD groups. *EID_FD* scan on energy-integrating detector computed tomography using full-dose protocol, *PCD-CT* scan on photon-counting detector computed tomography using full-dose protocol, *PCD_LD* scan on photon-counting detector computed tomography using low-dose protocol, *N. a.* not applicable

###  Objective Image Analysis

**Fig. 2:**
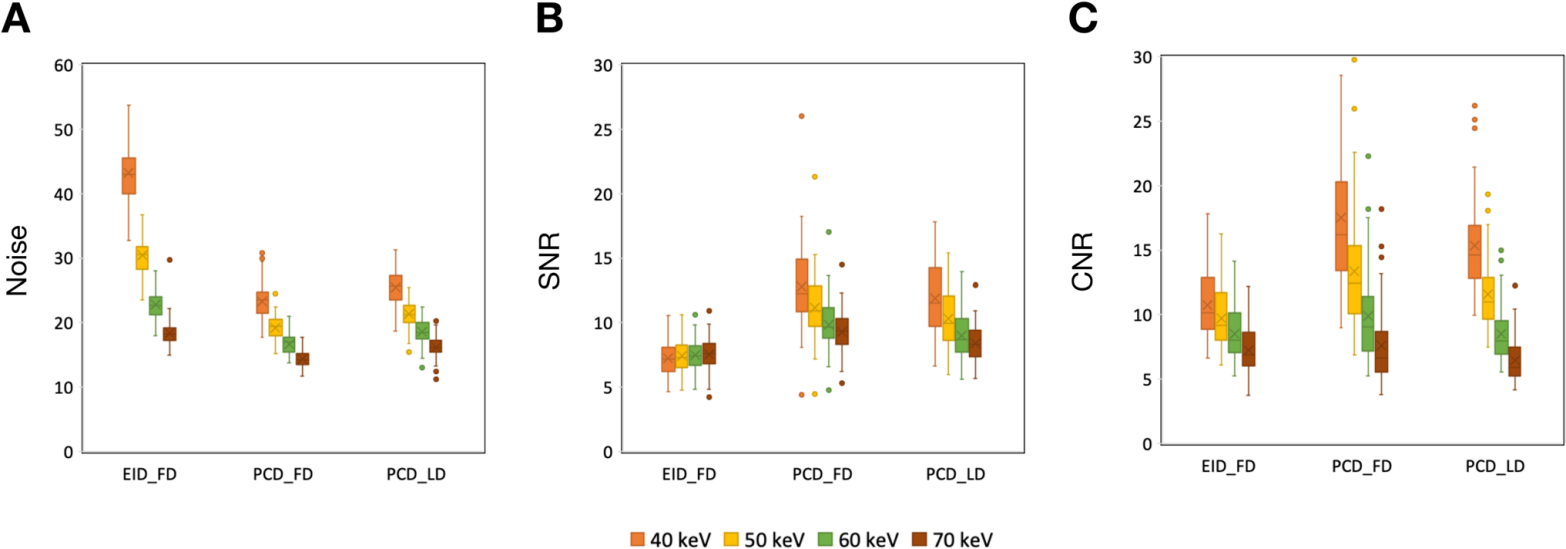
Boxplots of noise, SNR, and CNR values. SNR = signal-to-noise ratio; CNR, contrast-to-noise ratio; EID_FD, scan on energy-integrating detector computed tomography using full-dose protocol; PCD-CT, scan on photon-counting detector computed tomography using full-dose protocol; PCD_LD, scan on photon-counting detector computed tomography using low-dose protocol; SNR, signal-to-noise ratio; CNR, contrast-to-noise ratio; VMI, virtual monoenergetic imaging; keV, kiloelectron volt

**Fig. 3:**
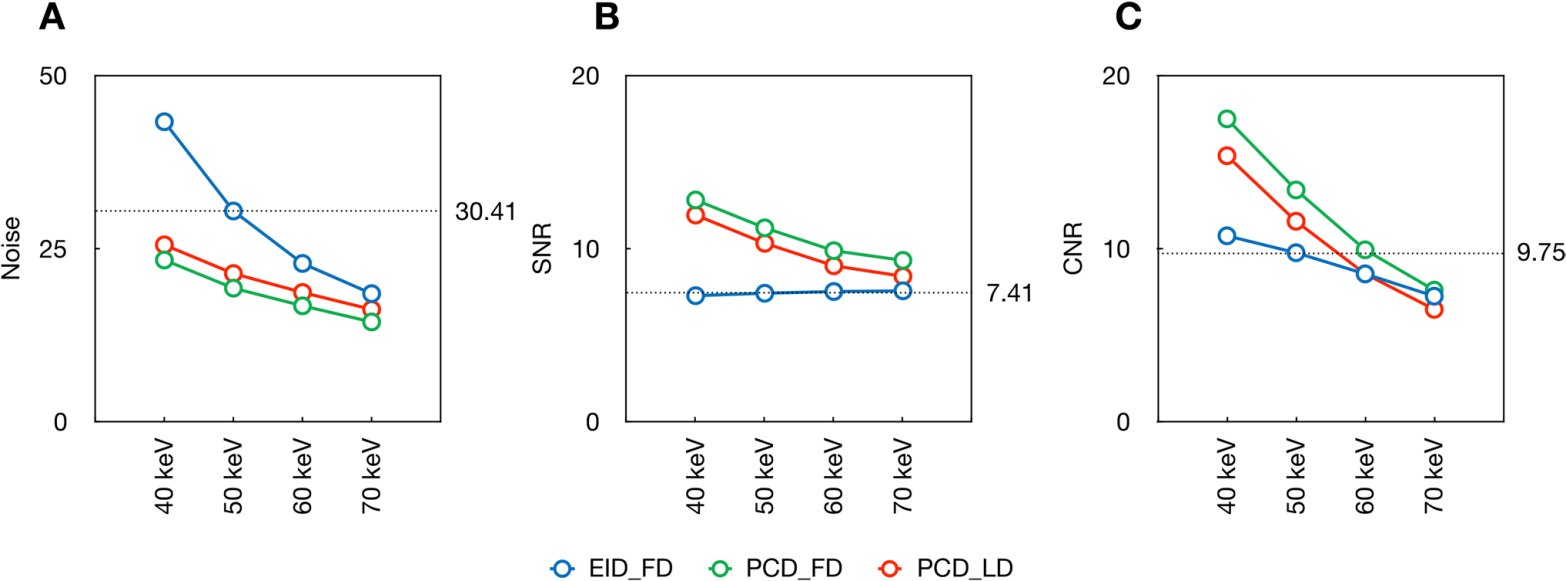
Line charts of noise, SNR, and CNR values. The reference line indicates the noise, SNR, and CNR values of the EID_FD VMI at 50 keV, which has been used in daily routine in our institution. SNR = signal-to-noise ratio; CNR = contrast-to-noise ratio; EID_FD = scan on energyintegrating detector computed tomography using full-dose protocol, PCD-CT = scan on photon-counting detector computed tomography using full-dose protocol, PCD_LD = scan on photon-counting detector computed tomography using low-dose protocol, SNR = signal-to-noise ratio, CNR = contrast-to-noise ratio, VMI = virtual monoenergetic imaging, keV = kiloelectron volt

The noise of VMI at all keV levels was significantly decreased by PCD_FD and PCD_LD compared to EID_FD (all adjusted *p* ≤ 0.002) (Table 3 and Fig. 2). The SNR values were significantly increased by PCD_FD and PCD_LD compared to EID_FD (all adjusted *p* ≤ 0.006). The CNR values were significantly increased by PCD_FD and PCD_LD compared to EID_FD only in VMI at 40 keV and 50 keV (all adjusted *p* ≤ 0.010). The PCD_LD presented higher noise (all adjusted *p* ≤ 0.002) and lower CNR values (all adjusted *p* ≤ 0.022) in VMI at all keV levels compared to PCD_FD, while the SNR values did not show significant difference between PCD_LD and PCD_FD in VMI at 40 keV and 50 keV (both adjusted *p* ≥ 0.058). Taking EID_FD VMI at 50 keV as reference, both PCD_FD and PCD_LD showed lower image noise and higher SNR and CNR with VMI at 50 keV and even at 40 keV (Fig. 3).

**Table 3:** HU, SD, SNR, and CNR values

	EID_FD (*n* = 59)	PCD_FD (*n* = 59)	PCD_LD (*n* = 59)	*p*	*p*1	*p*2	*p*3
Liver HU							
40 keV	313.13 ± 59.50	294.32 ± 61.66	300.75 ± 60.80	0.234	N. a	N. a	N. a
50 keV	224.50 ± 38.93	212.98 ± 40.36	217.64 ± 38.13	0.278	N. a	N. a	N. a
60 keV	170.57 ± 26.53	163.33 ± 27.61	166.13 ± 25.67	0.322	N. a	N. a	N. a
70 keV	137.28 ± 19.03	132.84 ± 19.77	134.53 ± 18.05	0.442	N. a	N. a	N. a
Liver SD							
40 keV	43.25 ± 4.40	23.33 ± 2.65	25.52 ± 2.92	< 0.001	< 0.001	< 0.001	0.002
50 keV	30.41 ± 2.89	19.26 ± 2.01	21.37 ± 2.18	< 0.001	< 0.001	< 0.001	< 0.001
60 keV	22.83 ± 2.05	16.71 ± 1.70	18.64 ± 1.81	< 0.001	< 0.001	< 0.001	< 0.001
70 keV	18.37 ± 2.13	14.38 ± 1.32	16.19 ± 1.58	< 0.001	< 0.001	< 0.001	< 0.001
Portal vein HU							
40 keV	773.90 ± 115.48	698.14 ± 147.58	687.95 ± 106.07	< 0.001	0.003	0.001	> 0.999
50 keV	519.34 ± 75.81	467.41 ± 95.19	463.32 ± 71.23	< 0.001	0.002	0.001	> 0.999
60 keV	364.59 ± 51.66	327.22 ± 64.53	324.12 ± 47.50	< 0.001	0.001	< 0.001	> 0.999
70 keV	269.15 ± 36.93	240.86 ± 45.70	238.59 ± 32.63	< 0.001	< 0.001	< 0.001	> 0.999
Liver SNR							
40 keV	7.27 ± 1.34	12.80 ± 3.26	11.93 ± 2.71	< 0.001	< 0.001	< 0.001	0.198
50 keV	7.41 ± 1.29	11.19 ± 2.54	10.30 ± 2.12	< 0.001	< 0.001	< 0.001	0.058
60 keV	7.51 ± 1.25	9.87 ± 1.98	9.00 ± 1.66	< 0.001	< 0.001	< 0.001	0.016
70 keV	7.55 ± 1.28	9.31 ± 1.62	8.39 ± 1.42	< 0.001	< 0.001	0.006	0.002
Liver-portal vein CNR							
40 keV	10.73 ± 2.44	17.49 ± 5.75	15.36 ± 3.72	< 0.001	< 0.001	< 0.001	0.019
50 keV	9.75 ± 2.24	13.38 ± 4.58	11.57 ± 2.70	< 0.001	< 0.001	0.010	0.011
60 keV	8.53 ± 1.99	9.92 ± 3.52	8.53 ± 2.07	< 0.001	0.014	> 0.999	0.014
70 keV	7.24 ± 1.84	7.59 ± 2.92	6.48 ± 1.67	0.023	> 0.999	0.192	0.022

Data were presented as mean ± standard deviation. *p p* value for one-way analysis of variance among the EID_FD, PCD_FD, and PCD_LD groups. *p*1 to *p*3 were *p* values for post hoc pairwise comparisons using Bonferroni correction; the *p* values presented were presented as adjusted *p* values. *p1 p* value between EID_FD and PCD_FD groups, *p2 p* value between EID_FD and PCD_LD groups, *p3 p* value between PCD_FD, PCD_LD groups. *EID_FD* scan on energy-integrating detector computed tomography using full-dose protocol, *PCD-CT* scan on photon-counting detector computed tomography using full-dose protocol, *PCD_LD* scan on photon-counting detector computed tomography using low-dose protocol, *keV* kiloelectron volt, *HU* Hounsfield unit, *SD* standard deviation, *SNR* signal-to-noise ratio, *CNR* contrast-to-noise ratio, *N. a.* not applicable

### Subjective Image Analysis

**Fig. 4:**
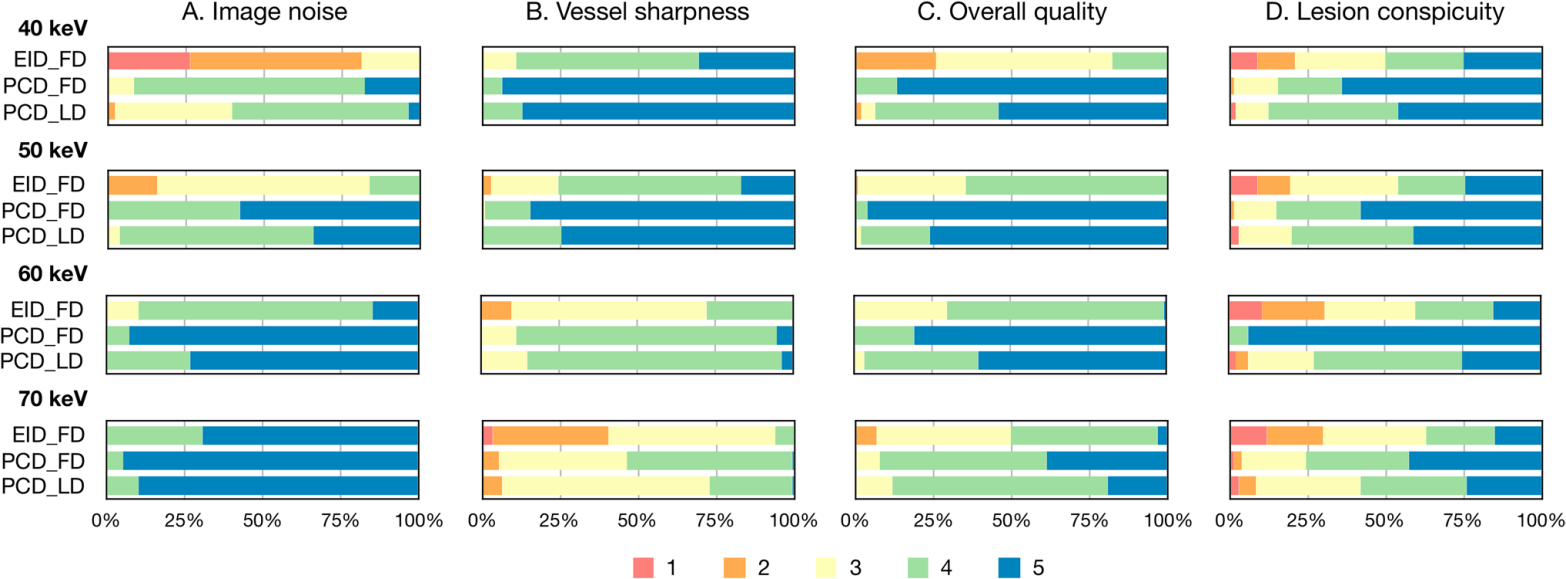
Bar plots of percentage of subjective ratings of image quality and hepatic lesion conspicuity Percentage of subjective ratings of (**A**) image noise, (**B**) vessel sharpness, (**C**) overall quality, and (**D**) lesion conspicuity. EID_FD = energy-integrating detector CT with full-dose protocol; PCD_FD = photon-counting detector CT with full-dose protocol; PCD_LD = photon-counting detector CT with low-dose protocol, keV = kiloelectron volt

The ratings of image noise, vessel sharpness, overall quality, and lesion conspicuity were significantly greater at all keV levels in PCD_FD and PCD_LD compared to EID_FD (all adjusted *p* ≤ 0.001) (Table 4 and Fig. 4). The PCD_FD showed higher ratings of image noise and overall quality in VMI at 40 to 60 keV compared to PCD_LD (all adjusted *p* ≤ 0.012), while the PCD_FD presented higher ratings of vessel sharpness only in VMI at 70 keV compared to PCD_LD (adjusted *p* = 0.019). There was no significant difference detected in the rating of lesion conspicuity between PCD_FD and PCD_LD in VMI at all keV levels (all adjusted *p* ≥ 0.259).

**Table 4:** Image quality and hepatic lesion conspicuity ratings

	EID_FD	PCD_FD	PCD_LD	*p*	*p*1	*p*2	*p*3
Image noise	*n* = 59	*n* = 59	*n* = 59				
40 keV	2.00 (1.50, 2.17)	4.00 (4.00, 4.00)	3.67 (3.00, 4.00)	< 0.001	< 0.001	< 0.001	< 0.001
50 keV	3.00 (2.67, 3.00)	4.67 (4.00, 5.00)	4.00 (4.00, 4.67)	< 0.001	< 0.001	< 0.001	0.009
60 keV	4.00 (4.00, 4.33)	5.00 (5.00, 5.00)	5.00 (4.33, 5.00)	< 0.001	< 0.001	< 0.001	0.008
70 keV	5.00 (4.33, 5.00)	5.00 (5.00, 5.00)	5.00 (5.00, 5.00)	< 0.001	< 0.001	0.001	> 0.999
Vessel sharpness	*n* = 59	*n* = 59	*n* = 59				
40 keV	4.00 (4.00, 4.67)	5.00 (5.00, 5.00)	5.00 (5.00, 5.00)	< 0.001	< 0.001	< 0.001	> 0.999
50 keV	4.00 (3.50, 4.33)	5.00 (5.00, 5.00)	5.00 (4.33, 5.00)	< 0.001	< 0.001	< 0.001	0.759
60 keV	3.00 (3.00, 3.33)	4.00 (4.00, 4.00)	4.00 (4.00, 4.00)	< 0.001	< 0.001	< 0.001	> 0.999
70 keV	2.67 (2.33, 3.00)	3.33 (3.00, 4.00)	3.00 (3.00, 3.33)	< 0.001	< 0.001	< 0.001	0.019
Overall quality	*n* = 59	*n* = 59	*n* = 59				
40 keV	3.00 (2.50, 3.33)	5.00 (5.00, 5.00)	4.67 (4.00, 5.00)	< 0.001	< 0.001	< 0.001	< 0.001
50 keV	4.00 (3.33, 4.00)	5.00 (5.00, 5.00)	5.00 (4.67, 5.00)	< 0.001	< 0.001	< 0.001	0.005
60 keV	4.00 (3.33, 4.00)	5.00 (5.00, 5.00)	5.00 (4.00, 5.00)	< 0.001	< 0.001	< 0.001	0.012
70 keV	3.33 (3.00, 4.00)	4.00 (4.00, 5.00)	4.00 (4.00, 4.00)	< 0.001	< 0.001	< 0.001	0.075
Lesion conspicuity	*n* = 35	*n* = 45	*n* = 33				
40 keV	3.67 (3.00, 4.33)	5.00 (4.00, 5.00)	4.33 (4.00, 5.00)	< 0.001	< 0.001	< 0.001	> 0.999
50 keV	3.33 (3.00, 4.33)	4.67 (4.00, 5.00)	4.33 (3.67, 5.00)	< 0.001	< 0.001	0.003	0.861
60 keV	3.33 (2.33, 4.00)	4.00 (3.67, 5.00)	4.00 (3.33, 4.33)	< 0.001	< 0.001	0.004	0.635
70 keV	3.33 (2.50, 4.00)	4.33 (3.67, 5.00)	4.00 (3.00, 4.33)	< 0.001	< 0.001	0.050	0.259

Data were presented as median (the first and the third quartile). *p p* value for Kruskal–Wallis H test among the EID_FD, PCD_FD, and PCD_LD groups. *p*1 to *p*3 were *p* values for post hoc pairwise comparisons using Bonferroni correction; the *p* values presented were presented as adjusted *p* values. *p1 p* value between EID_FD and PCD_FD groups, *p2 p* value between EID_FD and PCD_LD groups, *p3 p* value between PCD_FD, PCD_LD groups. *EID_FD* scan on energy-integrating detector computed tomography using full-dose protocol, *PCD-CT* scan on photon-counting detector computed tomography using full-dose protocol, *PCD_LD* scan on photon-counting detector computed tomography using low-dose protocol, *keV* kiloelectron volt

**Fig. 5:**
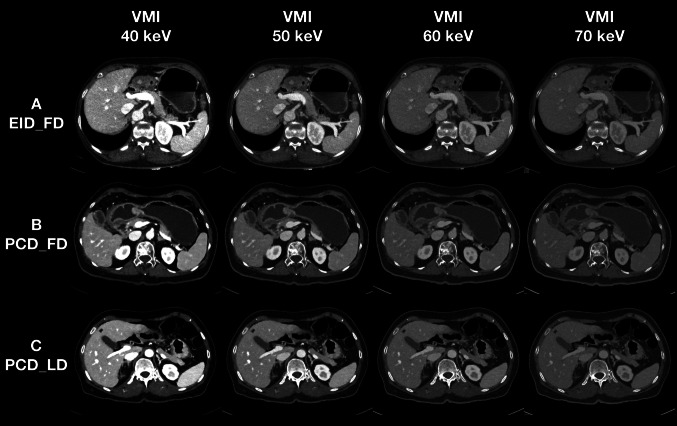
Representative cases for image quality assessment. Three matched cases of EID_FD, PCD_FD, PCD_LD scans of VMI at 40, 50, 60, and 70 keV for image quality assessment are presented with a window width of 800 HU and window level of 200 HU. (**A**) A 67-year-old female patient with a height of 1.59 m, a weight of 53.0 kg, and a body mass index of 20.94 kg/m^2^, who scheduled to undergo the contrast-enhanced abdominal CT scan for abdominal discomfort, on EID-CT with a full-dose protocol. (**B**) A 66-year-old female patient with a height of 1.56 m, a weight of 50.5 kg, and a body mass index of 20.74 kg/m^2^, who scheduled to undergo the contrast-enhanced abdominal CT scan before the endoscopy, on PCD-CT with a full-dose protocol. (**C**) A 66-year-old female patient with a height of 1.57 m, a weight of 50.0 kg, and a body mass index of 20.28 kg/m^2^, who scheduled to undergo the contrast-enhanced abdominal CT scan for suspicious gastric cancer, on PCD-CT with a low-dose protocol. The image noise increased with the decreasing of keV level of VMI, while the vessel sharpness became better with the decreasing of keV level of VMI. The image noise of EID_FD was higher than PCD_FD and PCD_LD, especially in VMI at 40 keV. The vessel sharpness was comparable between PCD_FD and PCD_LD. The PCD_FD and PCD_LD can provide better overall image quality than EID_FD. EID_FD = energyintegrating detector CT with full-dose protocol; PCD_FD = photon-counting detector CT with full-dose protocol; PCD_LD = photon-counting detector CT with low-dose protocol, VMI = virtual monoenergetic imaging, keV = kiloelectron volt

**Fig. 6:**
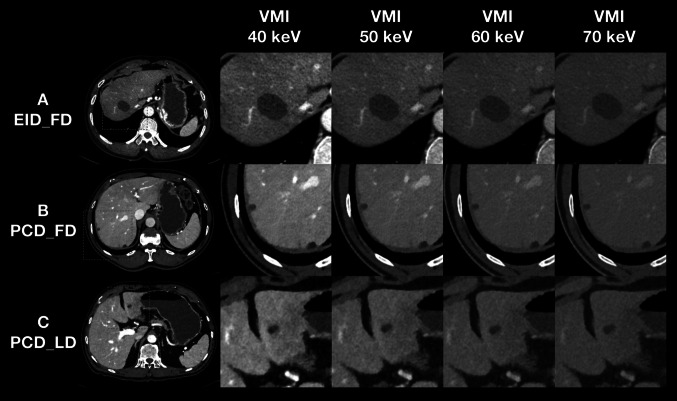
Representative cases for hepatic lesion conspicuity assessment. Three matched cases of EID_FD, PCD_FD, PCD_LD scans of VMI at 40, 50, 60, and 70 keV for hepatic lesion assessment are presented with a window width of 800 HU and window level of 200 HU. (**A**) A 59-year-old male patient with a height of 1.73 m, a weight of 72.0 kg, and a body mass index of 24.05 kg/m2, who scheduled to undergo the contrast-enhanced abdominal CT scan for heartburn and acid regurgitation, on EID-CT with a full-dose protocol. (**B**) A 61-year-old male patient with a height of 1.82 m, a weight of 79.0 kg, and a body mass index of 23.85 kg/m^2^, who scheduled to undergo the contrast-enhanced abdominal CT scan for abdominal pain, on PCD-CT with a full-dose protocol. (**C**) A 60-year-old male patient with a height of 1.72 m, a weight of 71.0 kg, and a body mass index of 24.00 kg/m^2^, who scheduled to undergo the contrast-enhanced abdominal CT scan for abdominal discomfort, on PCD-CT with a low-dose protocol. The contrast between the hypodense hepatic lesion and liver parenchyma and the border of the lesion became clearer with the decreasing of keV level of VMI, but the image noise got worse and potentially impacted on the lesion conspicuity. The lesion conspicuity of PCD_FD and PCD_LD benefited from the better overall image quality compared to EID_FD. EID_FD = energy-integrating detector CT with full-dose protocol; PCD_FD = photon-counting detector CT with full-dose protocol; PCD_LD = photon-counting detector CT with low-dose protocol, VMI = virtual monoenergetic imaging, keV = kiloelectron volt

For the overall image quality, most of EID_FD VMI at 50 keV was satisfied for clinical use (99.4%, 176/177), while more than three fourths of the EID_FD VMI at 40 keV (74.0%, 131/177) reached the average quality. In contrast, all the PCT_FD VMI at 40 keV (100.0%, 177/177) and most of the PCT_LD VMI at 40 keV (98.3%, 174/177) were acceptable for clinical use. Further, only a little of the PCT_FD VMI at 40 keV (1.5%, 2/135) PCT_LD VMI at 40 keV (2.0%, 2/99) were considered unacceptable in lesion conspicuity, while the corresponding percentage in EID_FD VMI at 40 keV was much higher (20.0%, 21/105). The representative cases for image quality and hepatic lesion conspicuity are presented (Figs. 5 and 6).

## Discussion

Our study found that the contrast-enhanced abdominal scan on PCD-CT with a low-dose protocol allows lower keV VMI with lower radiation dose and provides better image quality and lesion conspicuity than EID-CT in VMI at all evaluated keV levels. Further, the PCD-CT with a low-dose protocol shows comparable vessel sharpness and lesion conspicuity compared to PCD-CT with a full-dose protocol in low keV VMI, but the overall image quality slightly decreases due to the increased image noise.

It is the main reason for seeking lower keV VMI in contrast-enhanced abdominal CT scans that the low keV VMI can provide higher CNR values and better conspicuity of hypervascular lesions compared to conventional polychromatic images generated from single-energy CT [35–38]. Although the ideal keV level of VMI reconstruction of the portal-venous phase in the abdomen still highly depends on the diagnostic task [39–43], to create the VMI at keV levels as low as possible with acceptable image noise has been one of the most popular topics in abdominal CT scan. Different from PCD-CT, the EID-CT converts the X-ray photons first into light and then secondarily creates an electric signal for image reconstruction [11–13]. Due to the technical limitation of the EID-CT, the recent innovations of dual-energy EID-CT in low keV VMI reconstruction are mainly reconstruction or denoising algorithms [8–10, 44–46]. It is undeniable that the deep learning-based image reconstruction or denoising algorithms can reduce the radiation dose while keeping the image quality, but the rationale of deep learning-based image reconstruction algorithms is so far a black box, and there are still doubts about its potential influence on diagnostic purposes. In contrast, the PCD-CT can avoid the electronic noise in rationale [11–13], and therefore has the potential to allow lower keV VMI without additional advanced reconstruction algorithms. In addition, a phantom study has shown that PCD-CT can provide lower image noise and better spatial resolution, image texture, and detectability of simulated abdominal lesions at the same dose level compared to EID-CT [47]. Our study in human participants rhymed with the phantom study and further confirmed the potential for dose reduction for patients undergoing contrast-enhanced abdominal CT scans.

As a recently available CT technology, there is a limited number of literature regarding the use of contrast-enhanced abdominal VMI from PCD-CT [14–16]. All of the previous studies performed the scan with a tube voltage of 120 kVp [14–16], but our study selected 140 kVp to allow for better spectral separation in the abdomen as recommended by an expert’s consensus paper [48]. The other scan and reconstruction parameters also vary across the studies. Two studies used automatic tube current modulation at an image quality level of 145 [15, 16], which were the same as the PCD_FD scan in our study. On the other hand, one study chose to moderate the tube current to match the radiation dose of a previous EID-CT scan in order to achieve intra-individual dose neutrality [14]. These various scan parameters resulted in differences in the volume CT index and potentially influenced the image quality, as the image noise increases with the reduction of radiation dose. The PCD_FD scans in our study were performed with a radiation dose close to that of Higashigaito et al. [14] and lower than those of Bette et al. [15] and Estler et al. [16]. Our study found increased image noise in our PCD_LD scans compared to PCD_FD scans, but the image noise was still acceptable for diagnostic purposes. Our study has expanded the acceptable lower limit of radiation dose for contrast-enhanced abdominal scans by reducing about one third of the volume CT index of PCD_FD scans.

Further, the reconstruction parameters were also diverse in PCD-CT studies [14–16]. The slice thickness ranged from 1 to 3 mm with different slice increments. The strength levels of QIR were 2 to 3 out of 4, and the reconstruction kernels included Qr40, Br40f, and Br36. These differences also impact the image quality. We chose the highest strength level of QIR to fully present its potential in decreasing the image noise. We consider that future studies may select a high strength level of QIR to allow additional decrease of image noise while keeping the original image texture [28]. The extra allowance of image quality can translate to better lesion conspicuity or transform into radiation dose reduction. Our study used a reconstruction kernel of Br40, which is recommended by the vendor for image reconstruction of the portal-venous phase of contrast-enhanced abdominal scans, and may also slightly influence the image quality. We encourage future studies to use comparable reconstruction kernels to allow comparisons among studies.

We must acknowledge the following limitations of our study. First, our single-center study included a rather small number of participants. However, the post hoc power calculation guaranteed the robustness of our results. Although we have matched the patients according to sex, age, and body mass index, there were other confounding factors that potentially impact the image quality, such as their underlying medical conditions. As it is ethically not available to perform multiple scans within the same patient, future studies may consider a study design with a larger sample size and more rigorous matching criteria. Second, the scans on PCD-CT were performed with automatic adjustment of matrix and field of view, while the scans on EID-CT were with fixed settings. The image quality of PCD-CT might be further improved by using the patient-specific tailored protocol. Further, we used the same contrast media protocol for all the patients. The images could be more comparable among different image acquisition and reconstruction protocols if the contrast media injection is adjusted. Third, we compared only one strength level of the iterative reconstruction algorithm and one reconstruction kernel. The combination of the strength level and kernels with the keV level should be evaluated to optimize the protocol. Fourth, we only evaluated the radiation dose using volume CT dose index, dose-length product, and effective dose. The size-specific dose estimate was not applied as the required data were not automatically available on the EID-CT scanner. Fifth, the levels of reconstruction algorithms differ between EID-CT and PCD-CT systems. It is not clinically acceptable to use the highest level of the iterative reconstruction algorithm within EID-CT due to the waxy-like appearance, which could potentially mislead the radiologist's diagnosis. On the other hand, it is possible to reconstruct the images using a lower level of QIR. However, the comparison would be of nonsense as the level is not used in the clinical routine [28, 48]. Therefore, we selected the levels of reconstruction algorithms according to clinical practice to allow fair comparisons with clinical significance. Finally, the number of assessed hepatic lesions was small and mostly hypodense lesions. It is necessary to investigate the potential impact of the low-dose low-keV VMI on the diagnostic performance of specific abdominal lesions. We cannot reach a histological diagnosis because most of them are likely to be benign lesions. We agree with you that it is better to discuss the lesion conspicuity considering specific lesions. However, it is still fair to indicate the improvement of the lesions’ conspicuity as there are no significant differences between the lesions.

In conclusion, the contrast-enhanced abdominal scan with PCD-CT and a low-dose protocol reduced the radiation exposure and allows lower keV VMI to provide better image quality and lesion conspicuity than EID-CT in VMI at all evaluated keV levels, and acceptable lesion conspicuity compared to PCD-CT with a full-dose protocol in low keV VMI. The PCD-CT may enable VMI at 40 keV as a new clinical routine for contrast-enhanced abdominal CT scan with reduced radiation dose.

## Data Availability

The data that support the findings of this study are available from the corresponding author, upon reasonable request.

## Abbreviations

EID-CT: Energy-integrating detector computed tomography
PCD-CT: Photon-counting detector computed tomography
keV: Kiloelectron volt
VMI: Virtual monoenergetic imaging
FD: Full-dose
LD: Low-dose
SNR: Signal-to-noise ratio
CNR: Contrast-to-noise ratio
SD: Standard deviation
QIR: Quantum iterative reconstruction
ROI: Region of interest
HU: Hounsfield unit
